# The association between adherence to a plant-based diet and cognitive ageing

**DOI:** 10.1007/s00394-023-03130-y

**Published:** 2023-03-11

**Authors:** Annick P. M. van Soest, Ondine van de Rest, Renger F. Witkamp, Nathalie van der Velde, Lisette C. P. G. M. de Groot

**Affiliations:** 1 Division of Human Nutrition and Health, Wageningen University & Research, P.O. Box 12, 6700 AA Wageningen, The Netherlands; 2 Section of Geriatric Medicine, Department of Internal Medicine, Amsterdam UMC, University of Amsterdam, Amsterdam, The Netherlands; 3 Amsterdam Public Health, Aging and Later Life, Amsterdam, The Netherlands

**Keywords:** Plant-based diet, Omega-3 fatty acids, Cognition, Older adults, Elderly, Healthy ageing

## Abstract

**Purpose:**

While the benefits of adopting a more plant-based diet for sustainability and animal welfare are clear, its long-term health impacts, including the impact on cognitive ageing, are limited studied. Therefore, we investigated the associations between plant-based diet adherence and cognitive ageing.

**Methods:**

Data from a previous intervention study involving community-dwelling adults aged ≥ 65 years were analysed at baseline (n = 658) and after 2-year follow-up (n = 314). Global and domain-specific cognitive functioning were assessed at both timepoints. Overall, healthful and unhealthful plant-based dietary indices were calculated from a 190-item food frequency questionnaire. Multivariate-adjusted linear regression models were applied to test for associations.

**Results:**

After full-adjustment, higher overall adherence to a plant-based diet was not associated with global cognitive function (difference in Z-score, tertile 1 versus 3 [95% CI]: 0.04 [− 0.05, 0.13] p = 0.40) or cognitive change (− 0.04 [− 0.11, 0.04], p = 0.35). Similarly, healthful and unhealthful plant-based diet indices were not associated with cognitive functioning (respectively p = 0.48; p = 0.87) or change (respectively p = 0.21, p = 0.33). Interestingly, we observed fish consumption to influence the association between plant-based diet adherence and cognitive functioning (p-interaction = 0.01), with only individuals with a fish consumption of ≥ 0.93 portion/week benefitting from better overall plant-based diet adherence (β per 10-point increment [95% CI]: 0.12 [0.03, 0.21] p = 0.01).

**Conclusion:**

We did not demonstrate associations of a more plant-based diet with cognitive ageing. However, possibly such association exists in a subpopulation with higher fish intake. This would be in line with earlier observations that diets rich in plant foods and fish, such as the Mediterranean diet, may be beneficial for cognitive ageing.

**Trial registration:**

Registered at clinicaltrials.gov (NCT00696514) on June 12, 2008.

**Supplementary Information:**

The online version contains supplementary material available at 10.1007/s00394-023-03130-y.

## Introduction

Consumers are increasingly opting for more plant-based diets, for various reasons related to sustainability, animal welfare and presumed health benefits. Nevertheless, the evidence supporting health benefits of shifting to a plant-based diet remains limited. While protective associations have been demonstrated for cardiovascular disease [1], cancer [2], and diabetes [3], little is known about the long-term effects of a shift towards a more plant-based diet on healthy ageing, including the effect on cognitive abilities.

At the same time, there is emerging evidence for beneficial effects of individual plant-derived components and foods on cognitive ageing. For example, higher consumption of polyphenols, vitamins C and E, carotenoids and unsaturated fatty acids, and plant foods rich in these plant-derived components, including vegetables, berries, nuts, olive oil, tea and coffee, have been associated to favourable brain ageing outcomes [4]. Similarly, higher adherence to the Mediterranean diet (MedDiet) and the Mediterranean-Dietary Approaches to Stop Hypertension Intervention for Neurodegenerative Delay (MIND) diet has been associated with better cognitive performance and slower rates of cognitive decline [5]. These dietary patterns, though not exclusively plant-based, are plant-centred and rich in brain-health promoting plant-derived components and foods.

Whereas these plant-derived components, foods and plant-centred dietary patterns have been shown to contribute to healthy cognitive ageing, there is no direct evidence to support the benefits of higher adherence to a plant-based dietary pattern. The few preliminary studies on the role of a more plant-based or vegetarian diet show promising positive associations with cognitive ageing outcomes [6–9], though not all studies support these findings [10, 11]. This merits further research on the role of a more plant-based diet on cognitive ageing.

To this end, the current study aims to investigate the association between plant-based diet adherence and cognitive functioning and 2-year cognitive decline in cognitively healthy older adults. To categorize plant-based diet adherence, we used the approach proposed by Satija et al. [12] based on an overall, healthful and unhealthful plant-based dietary index.

## Methods

### Participants and study design

The present study made use of data from the B-vitamins for the Prevention of Osteoporotic Fractures (B-proof) trial [13], a randomized double-blind placebo-controlled trial on the effect of 2 year supplementation with B-vitamins in community-dwelling adults aged ≥ 65 years with elevated homocysteine levels (12–50 μmol/L) on fracture incidence. Cognition was measured pre- and post-intervention as secondary outcome. The intervention existed of B-vitamin supplementation (400 μg folic acid and 500 μg vitamin B12) versus placebo. Participants did not suffer from renal insufficiency (creatinine > 150 μmol/L) and did not have a diagnosis of a malignancy in the past 5 years. For the present study, we used baseline data to perform a cross-sectional analysis. Next to this we performed a longitudinal analysis using follow-up data from the control group only, in order to eliminate any influence of the B-vitamin intervention, as previous research has demonstrated that this intervention may have slowed down cognitive decline in a subpopulation [14]. Data were collected between October 2008 and March 2013 in three research centres in the Netherlands: Erasmus Medical Center (Rotterdam), VU University Medical Center (Amsterdam) and Wageningen University (Wageningen). The current analysis is based on the Wageningen participants, as only this subpopulation underwent extensive cognitive testing (n = 856) and completed a food frequency questionnaire (FFQ) (n = 664). Of these 664 participants, data from 6 participants were excluded due to unreliable energy intake data (for men < 800 kcal or > 4200 kcal, women < 500 kcal or > 3500 kcal, n = 2) or due to missing baseline cognition data (n = 4). The final study sample comprised 658 participants*.* The study was approved by the Medical Ethics committee of Wageningen University & Research and has been registered at clinicaltrials.gov as NCT00696514. All participants had given written informed consent.

### Dietary assessment

Habitual dietary intake was assessed at baseline by a 190-item FFQ, of which validity has been reported previously [15, 16]. Participants were asked how often they had consumed a food item in the past month. Portion sizes were estimated using standard portion sizes and commonly used household measures. Average daily nutrient intakes were calculated based on the Dutch food composition database [17].

As a measure of plant-based diet adherence, we calculated the overall, healthful and unhealthful plant-based diet index (PDI, hPDI, uPDI, respectively) [12]. These indices included a total of 18 food groups, of which 7 are designated as healthy plant-based groups (whole grains, fruits, vegetables, nuts, legumes, vegetable oils, and tea & coffee), 5 as unhealthy plant-based groups (fruit juices, refined grains, potatoes, sugar-sweetened beverages, and sweets) and 6 classified as animal-based food groups (animal fat, dairy, fish, meat, eggs, and miscellaneous animal-based) (Supplementary Table 1). For scoring of the indices, intake of each food group was ranked into cohort-specific quintiles and each quintile was assigned a score ranging from 1 to 5. In the PDI (overall), both healthy and unhealthy plant-based groups were scored positively (i.e. higher intakes received higher scores). For the hPDI, healthy plant food groups were given positive scores and unhealthy plant-foods received reversed scores. For the uPDI, healthy plant-foods received reversed scores and unhealthy plant foods received positive scores. Animal-based food groups were scored reversely in all three indices. The 18 food group quintile scores were summed to obtain the index scores. Alcohol and margarine intake were not included in the indices but adjusted for in the analysis, in line with previous research [12]. Furthermore, the diet indices were adjusted for energy intake using the residual method [18].

### Cognitive testing

Cognitive functioning was assessed by trained research assistants with an extensive battery of cognitive tests at baseline and after 2 years. This battery included the Rey Auditory Verbal Learning Test (RAVLT) (subtests immediate, delayed and recognition) [19], the Digit Span task [20], the Trail Making Test (TMT) (part A and B) [21], the Stroop Colour-Word test (part I, II and III) [22], the Symbol Digit Modalities Test (SDMT) [23], and Letter Fluency [24] (Supplementary Table 2). Parallel versions were used for RAVLT, TMT and letter fluency to reduce learning effects.

To limit the number of cognition outcomes, cognitive composite scores were created. Individual cognitive test scores at baseline and after 2 yeras were converted into Z-scores based on population mean and standard deviation at baseline. The Z-scores for the TMT and Stroop Colour-Word test were reversed as lower scores represent better cognitive functioning. Individual Z-scores per test were clustered into composite scores for global and domain-specific cognitive functioning.

$$ Global cognition = \left( {Z_{Episodic memory} + Z_{Attention \& working memory} + Z_{Information processing speed} + Z_{Executive functioning} } \right)/4 $$

$$ Episodic memory = \left( {Z_{RAVLT immediate} + Z_{RAVLT delayed} + Z_{RAVLT recognition} } \right)/3 $$

$$ Attention \& working memory = \left( {Z_{Digit span forward} + Z_{Digit span backward} } \right)/2 $$

$$ Information processing speed = \left( { - Z_{Stroop mean I and II } + - Z_{TMT part A} + Z_{SDMT} } \right)/3 $$

$$ Executive functioning = \left( { - Z_{Stroop interference} + - Z_{TMT part B/A} + Z_{Fluency} } \right)/3 $$

Finally, Mini-Mental State Examination [25] was administered following standardized procedures. This score was measured for descriptive purposes (as an indicator of the cognitive state of the participants) rather than as outcome variable.

### Covariates

Information on age, gender, education level (low, middle, high), smoking status (never, former, current) was collected via questionnaires. Body weight and height were measured by trained research assistants. Body mass index (BMI) was calculated as weight (kg)/(height (m))^2^. Physical activity was assessed using the LASA physical activity questionnaire [26], and expressed in metabolic equivalent hours per week (MET h/w) covering activities of walking, cycling, sports, gardening and housework. Alcohol and margarine intake were derived from the FFQ.

### Statistical analysis

Data are expressed as n (%), mean (SD) or median (IQR) unless otherwise stated. Participant characteristics between PDI tertiles were compared using ANOVA or Kruskal–Wallis test for continuous variables, and chi-square test for categorical variables. Multiple linear regression analyses were performed to investigate the association between plant-based diet adherence and cognition. For the cross-sectional analysis, we modelled cognitive function at baseline as a function of plant-based diet adherence (PDI, hPDI and uPDI, in tertiles and continuous), using data of the total study population. For the longitudinal analysis, the change in cognition Z-score between baseline and after 2 years was modelled as a function of plant-based diet adherence (PDI, hPDI and uPDI, in tertiles and continuous). Here, only data from the control group were used, to eliminate interference of the B-vitamin intervention. All analyses were adjusted for age (in years), gender, education level (low, middle, high), BMI (in kg/m^2^), physical activity (in MET h/w), smoking (never, current, former), alcohol intake (light, moderate, excessive), and margarine intake (portions/d). The longitudinal analysis was additionally adjusted for baseline cognition Z-scores. To investigate if consumption of specific animal food groups modified the association, stratified analyses by fish, meat, egg and dairy consumption (median split) were performed. P-values smaller than 0.05 were considered significant. All analyses were performed using RStudio Version 1.4.0 [27].

## Results

### Baseline characteristics

Participant characteristics are shown in Table 1. The mean age at baseline was 72.1 ± 5.4 years, and 59% was male. On average, participants were overweight with a mean BMI of 27.2 ± 3.9 kg/m^2^ and cognitively healthy as indicated by a median MMSE score of 29 [28–30]. Participants who fell into the lowest tertile with respect to their plant-based diet adherence were on average more often male (p = 0.02), had a higher BMI (p < 0.01) and consumed more alcohol (p < 0.01) compared to individuals classified in the tertile with highest adherence. Nutrient intake differed between plant-based diet adherence tertiles (Supplementary Table 3). Participants with higher adherence to a plant-based diet had higher intakes of carbohydrates, sugar, fibre, and folic acid, while their intakes of protein, EPA, DHA and vitamin B12 were lower.

**Table 1:** Participant characteristics according to overall plant-based diet index tertiles

Characteristic	Overall (n = 658)	Tertile 1 (n = 226)	Tertile 2 (n = 202)	Tertile 3 (n = 230)	p-value
PDI score	54.0 ± 6.3	47.2 ± 3.4	54.0 ± 1.4	60.7 ± 3.1	< 0.001
Age (years)	72.1 ± 5.4	71.8 ± 5.2	72.3 ± 5.6	72.3 ± 5.3	0.59
Sex *n* (%)					0.02
Male	391 (59%)	151 (67%)	110 (54%)	130 (57%)	
Female	267 (41%)	75 (33%)	92 (46%)	100 (43%)	
Level of education *n* (%)					0.42
Low	280 (43%)	103 (46%)	50 (39%)	99 (43%)	
Middle	157 (24%)	50 (22%)	47 (23%)	60 (26%)	
High	221 (34%)	73 (32%)	77 (38%)	71 (31%)	
Ethnicity *n* (%)					
White	624 (95%)	217 (96%)	192 (95%)	215 (93%)	0.06
Asian	25 (4%)	3 (1%)	10 (5%)	12 (5%)	
Unknown	9 (1%)	6 (3%)	0 (0%)	3 (1%)	
BMI (kg/m^2^)	27.2 ± 3.9	28.1 ± 3.6	27.2 ± 4.5	26.3 ± 3.4	< 0.001
Physical activity (MET h/w)	53.4 [33.4–79.8]	51.5 [31.5–76.4]	53.1 [34.1–79.4]	56.7 [35.4–85.5]	0.15
Smoking behavior *n* (%)					0.19
Never smoker	200 (30%)	56 (25%)	70 (35%)	74 (32%)	
Current smoker	70 (11%)	27 (12%)	22 (11%)	21 (9%)	
Former smoker	388 (59%)	143 (63%)	110 (54%)	135 (59%)	
Alcohol consumption					< 0.001
Light	424 (64%)	115 (51%)	135 (67%)	174 (76%)	
Moderate	213 (32%)	101 (45%)	59 (29%)	53 (23%)	
(Very) excessive	21 (3%)	10 (4%)	8 (4%)	3 (1%)	
Margarine intake (portion/d)	15.4 [5.1–27.9]	14.9 [5.3–28.2]	12.8 [3.3–24.6]	18.8 [6.0–31.1]	0.01
MMSE score	29 [28–30]	29 [27–30]	29 [28-30]	29 [28-30]	0.29

*PDI* plant-based diet index, *BMI* body mass index, *MMSE* Mini Mental State Examination

Data are mean ± SD, median (IQR) or number (%)

### Cross-sectional analysis

In the fully adjusted models, a higher overall adherence to a plant-based diet, as well as higher adherence to either a healthful or an unhealthful plant-based diet were not associated with global cognitive functioning (difference tertile 1 vs 3 [95% CI]: PDI 0.04 [− 0.05, 0.13] p = 0.40; hPDI − 0.03 [− 0.13, 0.06] p = 0.48; uPDI − 0.01 [− 0.10, 0.09] p = 0.87) (Table 2). With respect to domain-specific cognitive functioning, individuals with a higher overall adherence to a plant-based diet showed better episodic memory compared to individuals with lower overall plant-based diet adherence (difference tertile 1 vs 3: 0.16 [0.03, 0.28], p = 0.01) (Supplementary Table 4). However, this finding was not confirmed in the continuous analysis (p-trend = 0.08). For the remaining three cognitive domains, no associations were found between overall, healthful or unhealthful plant-based diet adherence and attention & working memory, information processing speed, or executive functioning.

**Table 2:** Regression output energy-adjusted overall, healthful and unhealthful plant based diet index and global cognitive functioning (cross-sectional) and change (longitudinal)

	PDI			hPDI			uPDI		
	Crude model	Model 1	Model 2	Crude model	Model 1	Model 2	Crude model	Model 1	Model 2
Cross-sectional									
Tertile 1	REF	REF	REF	REF	REF	REF	REF	REF	REF
Tertile 2	0.11 [0.01, 0.22] 0.03	0.07 [– 0.03, 0.16] 0.16	0.09 [– 0.01, 0.18] 0.07	0.06 [– 0.04, 0.16] 0.26	0.03 [– 0.06, 0.12] 0.52	0.03 [– 0.06, 0.12] 0.52	– 0.03 [– 0.14, 0.07] 0.55	0.00 [– 0.09, 0.09] 0.98	0.00 [– 0.09, 0.09] 0.98
Tertile 3	0.04 [– 0.06, 0.15] 0.40	0.03 [– 0.06, 0.12] 0.56	0.04 [– 0.05, 0.13] 0.40	0.07 [– 0.03, 0.18] 0.16	– 0.01 [– 0.10, 0.08] 0.81	– 0.03 [– 0.13, 0.06] 0.48	– 0.14 [– 0.24, – 0.04] 0.01	– 0.02 [– 0.12, 0.07] 0.65	– 0.01 [– 0.10, 0.09] 0.87
Continuous	0.05 [– 0.02, 0.12] 0.14	0.03 [– 0.03, 0.09] 0.31	0.04 [– 0.02, 0.10] 0.22	0.06 [– 0.01, 0.12] 0.08	0.00 [– 0.06, 0.05] 0.87	– 0.02 [– 0.08, 0.03] 0.46	– 0.08 [– 0.15, – 0.02] 0.01	– 0.02 [– 0.07, 0.04] 0.55	– 0.01 [– 0.06, 0.05] 0.83
Longitudinal									
Tertile 1	REF	REF	REF	REF	REF	REF	REF	REF	REF
Tertile 2	– 0.09 [– 0.16, – 0.01] 0.02	– 0.08 [– 0.15, 0.00] 0.04	– 0.08 [– 0.16,– 0.01] 0.03	0.00 [– 0.08, 0.07] 0.89	0.00 [– 0.07, 0.07] 0.99	0.00 [– 0.07, 0.07] 0.92	– 0.03 [– 0.10, 0.04] 0.42	– 0.03 [– 0.11, 0.04] 0.35	– 0.03 [– 0.10, 0.05] 0.47
Tertile 3	– 0.02 [– 0.09, 0.05] 0.65	– 0.01 [– 0.08, 0.06] 0.73	– 0.04 [– 0.11, 0.04] 0.35	0.06 [– 0.01, 0.14] 0.10	0.06 [– 0.01, 0.13] 0.11	0.05 [– 0.03, 0.12] 0.21	– 0.03 [– 0.11, 0.04] 0.42	– 0.03 [– 0.11, 0.04] 0.41	– 0.04 [– 0.11, 0.04] 0.33
Continuous	– 0.01 [– 0.06, 0.04] 0.73	– 0.01 [– 0.06, 0.04] 0.70	– 0.03 [– 0.08, 0.02] 0.30	0.04 [0.00, 0.08] 0.08	0.04 [0.00, 0.09] 0.06	0.03 [– 0.01, 0.08] 0.17	– 0.04 [– 0.08, 0.01] 0.09	– 0.04 [– 0.09, 0.00] 0.06	– 0.05 [– 0.09, 0.00] 0.04

Model 1: adjusted for age, gender and education. Model 2: additionally adjusted for BMI, physical activity, smoking, alcohol consumption and margarine consumption. Longitudinal analysis was additionally adjusted for baseline cognition score

*PDI* plant-based diet index, *hPDI* healthful plant-based diet index, *uPDI* unhealthful plant-based diet index

Data are β [95% CI] p-value. In the continuous analysis, β is shown per 10 points increment in plant-based diet index

### Longitudinal analysis

Higher adherence to an overall or healthful plant-based diet was not associated with the rate of cognitive decline over 2 years (difference tertile 1 vs 3 [95% CI]: PDI -0.04 [-0.11, 0.04], p = 0.35; hPDI 0.05 [− 0.03, 0.12] p = 0.21) (Table 2). Individuals with the highest adherence to an unhealthful plant-based diet did not show steeper rates of cognitive decline compared to those with lowest adherence (difference T1 vs T3: − 0.04 [− 0.11, 0.04], p = 0.33), though the continuous analysis indicated a significant trend (β per 10-point increment: − 0.05 [− 0.09, 0.00], p = 0.04).

With respect to domain-specific cognitive functioning, attention & working memory was influenced by the degree of adherence to a plant-based diet (Supplementary Table 5). Better adherence to a healthful plant-based diet was associated with slower rates of cognitive decline in attention & working memory (difference tertile 1 vs 3: 0.23 [0.05, 0.41], p = 0.01, p-trend = 0.01), while higher unhealthful plant-based diet adherence was associated with faster rates of decline (difference tertile 1 vs 3: − 0.18 [− 0.36, − 0.01], p = 0.04, p-trend < 0.01). Overall adherence to a plant-based diet was not associated with a decline in attention & working memory (p-trend = 0.29).

We did not find associations between the plant-based dietary indices and episodic memory, information processing speed, or executive functioning.

### Sensitivity analysis

To investigate if consumption of specific animal food groups modified the association between adherence to a plant-based diet and cognitive ageing, we performed stratified analyses by fish, meat, egg and dairy consumption based on a median split.

For the sensitivity analysis stratified by fish intake, participants were divided into two groups, those with lower and higher fish intake than the median fish intake of 0.93 portion per week. Interestingly, fish consumption appeared to influence the association between adherence to a plant-based diet and cognition (Table 3). Cross-sectionally, higher overall plant-based diet adherence was associated with better global cognitive functioning in individuals with higher fish consumption (β per 10-point increment 0.12 [0.03, 0.21], p = 0.01), while in individuals with lower fish consumption no association was observed (β per 10-point increment − 0.03 [− 0.12, 0.06], p = 0.52; p-interaction = 0.01). Longitudinally, the association between the rate of cognitive change with healthful plant-based diet adherence appeared to be modified by fish consumption in a similar manner (p-interaction < 0.01): higher healthful plant-based diet adherence was associated with slower rates of cognitive decline in individuals with higher fish consumption (0.07 [0.00, 0.14], p = 0.04), but not in those with lower fish consumption (− 0.02 [− 0.08, 0.04], p = 0.56). The association between overall plant-based adherence and cognitive decline appeared to be influenced by fish consumption as well (p-interaction < 0.01), but in the opposite direction. We did not find an association between overall adherence to a plant-based diet and cognitive decline in individuals with higher fish consumption (0.05 [− 0.04, 0.13], p = 0.27), while a negative association became apparent in individuals with lower fish consumption (− 0.10 [− 0.16, − 0.03], p < 0.01). This interaction in opposite direction was solely driven by the lower episodic memory performance of the individuals with lower fish intake, and was not observed for the other cognitive domains (data not shown).

**Table 3:** Association between overall, healthful and unhealthful plant-based diet index and cognitive functioning and change, stratified by fish consumption

	PDI				hPDI				uPDI			
	Effect size			Overall interaction	Effect size			Overall interaction	Effect size			Overall interaction
	Crude	Adjusted	p-value	p-value	Crude	Adjusted	p-value	p-value	Crude	Adjusted	p-value	p-value
Cross-sectional												
Fish intake < 0.93 portion/w	– 0.02 [– 0.12, 0.07]	– 0.03 [– 0.12, 0.06]	0.52	0.01	0.03 [– 0.06, 0.12]	– 0.04 [– 0.12, 0.05]	0.38	0.44	– 0.09 [– 0.18, 0.00]	– 0.02 [– 0.11, 0.06]	0.57	0.58
Fish intake ≥ 0.93 portion/w	0.13 [0.03, 0.23]	0.12 [0.03, 0.21]	0.01		0.08 [0.00, 0.16]	0.01 [– 0.07, 0.09]	0.77		– 0.09 [– 0.18, 0.01]	0.00 [– 0.09, 0.09]	0.97	
Longitudinal												
Fish intake < 0.93 portion/w	– 0.08 [– 0.13, – 0.02]	– 0.10 [– 0.16, – 0.03]	< 0.01	< 0.01	– 0.03 [– 0.08, 0.03]	– 0.02 [– 0.08, 0.04]	0.56	< 0.01	– 0.01 [– 0.06, 0.05]	– 0.02 [– 0.08, 0.04]	0.52	0.34
Fish intake ≥ 0.93 portion/w	0.06 [– 0.02, 0.15]	0.05 [– 0.04, 0.13]	0.27		0.10 [0.03, 0.17]	0.07 [0.00, 0.14]	0.04		– 0.07 [– 0.15, 0.00]	– 0.07 [– 0.15, 0.01]	0.08	

Adjusted model was adjusted for age, gender and education, BMI, physical activity, smoking, alcohol consumption and margarine consumption. *PDI* plant-based diet index, *hPDI* healthful plant-based diet index, *uPDI* unhealthful plant-based diet index

Longitudinal analysis was additionally adjusted for baseline cognition score. Data are β [95% CI] and β is shown per 10 points increment in plant-based diet index

We did not find proof for modification by the other animal food groups, i.e. meat, egg, or dairy (data not shown).

## Discussion

In this cohort of Dutch cognitively healthy older adults, there was no evidence for a beneficial association between adherence to a plant-based diet and cognitive ageing. While individuals who adhered better to a plant-based diet consumed more fibre and less cholesterol and saturated fatty acids, their intakes of vitamin B12, EPA and DHA were lower*.* Interestingly, a higher consumption of fish, rich in the latter nutrients, appeared to partly influence the association between adherence to a plant-based diet and cognitive ageing.

To our knowledge, the association between the degree of adherence to a plant-based or vegetarian diet with cognitive ageing has been investigated in six other studies, with mixed results. Three studies demonstrated positive associations: a more plant-based dietary pattern as derived from principle component analysis was associated with better cognitive functioning in older adults [7], and higher scores on the overall and healthful plant-based diet index were associated with a lower risk of cognitive impairment in two Asian cohorts [6, 9]. At the same time, mixed results were observed in an American study [8]. Here, higher adherence to a healthful plant-based diet was associated with slower rates of decline in different cognitive domains in older African Americans, but no association was observed for White Americans in this same cohort. A null-finding comes from a small sample of non-demented community dwelling older adults, in which vegetarians did not perform better on cognitive tests or had lower odds of mild cognitive impairment compared to omnivores [11]. In addition, a higher pro-vegetarian score was not associated with 6 year change in Telephone Interview of Cognitive Status (TICS) scores in middle-aged to older adults [10]. The reason for the inconsistency in findings is hard to pinpoint, as comparability is limited due to differences in study population, duration of follow-up, exposure variable and outcome measures. Importantly, even studies that make use of the plant-based diet index as exposure variable cannot be compared directly, as this index makes use of population-specific cut-offs. An important limitation of our analysis that could be responsible for our null-finding is the duration of follow-up. Two years is relatively short to detect cognitive decline in cognitively healthy older individuals. Nevertheless, we used an extensive cognitive test battery to be able to capture subtle cognitive deteriorations rather than the more general MMSE or TICS. Furthermore, the degree of adherence to a plant-based diet was only determined at baseline. However, we do assume that our measurement represents long-term intake as dietary patterns in the elderly are fairly stable over time [28].

Whatever the true association between plant-based diet adherence and cognitive ageing may be, the lack of beneficial association in our analysis can be explained from a nutrient perspective. A diet rich in plant foods contains many nutrients that are beneficial for healthy brain ageing, including vitamin C, vitamin E, polyphenols, carotenoids and unsaturated fatty acids. These nutrients have demonstrated anti-oxidant and anti-inflammatory properties, via which they could slow down cognitive decline during ageing [4]. However, a diet predominantly containing plant foods may be lacking some crucial nutrients for optimal brain functioning, including vitamin B12, EPA and DHA. Vitamin B12, in conjunction with vitamin B6 and folic acid, plays an important role in regulating homocysteine levels, an important risk factor for cognitive decline and dementia [29]. EPA and DHA are involved in different mechanisms shown to be important to maintain brain health. For example, these long chain omega-3 polyunsaturated fatty acids are important building blocks of brain tissue, and have anti-inflammatory, anti-oxidative and vascular health promoting effects [30].

In our sensitivity analysis, we found that fish consumption modified the association between adherence to a plant-based diet and cognition, with only individuals with a higher fish consumption seeming to benefit from adhering to a plant-based diet. While fish, rich in vitamin B12, EPA and DHA, has been shown to slow cognitive decline on its own [31], combining fish with a diet rich in plant foods may have additional benefits. A multi-nutrient approach seems crucial for healthy brain ageing, as the mechanisms underlying nutrition and brain ageing are multifactorial [32]. This is also evidenced by the stronger evidence for dietary patterns versus single nutrients or foods [4] and the synergistic effect of omega-3 fatty acids and anti-oxidants [33]. Alternatively, the modification by fish may be explained by a shift in of animal-based product consumption, i.e. from meat to fish. Meat is an important source of saturated fatty acids, which have been associated to worse cognitive functioning and higher risks of mild cognitive impairment and dementia [4]. In addition, various dietary patterns low in meat have been associated with favourable brain ageing outcomes [5]. However, observational studies on the association between meat intake and cognitive ageing mostly demonstrate no associations [34], thus direct evidence for a possible negative effect of meat is lacking.

Observational studies can confirm the combined beneficial associations of a diet rich in plant foods and fish in cognitive ageing. The MedDiet, a diet rich in vegetables, fruits, whole grains, nuts and fish, has been associated with better cognitive functioning, slower rates of cognitive decline and lower chance of dementia [5]. Similar benefits have been demonstrated for the MIND diet, which composition is based on the MedDiet but with emphasis on the specific brain foods such as berries and leafy greens [5]. In addition, a study into the association between the plant-based diet index and cognitive ageing showed that plant-based dietary patterns including fish were more protective against risk of cognitive impairment compared to plant-based dietary patterns without fish [9].

While the modification by fish intake can be explained from a nutrient perspective and observational studies support this finding, it needs to be mentioned that our findings result from a subgroup analysis which limits the interpretability of these observations. Possibly, this is also an explanation for the inconsistency in findings, as we only demonstrated the modification by fish intake for PDI in the cross-sectional analysis, and hPDI in the longitudinal analysis. These results should be interpreted as preliminary and the analyses have to be replicated in other datasets before definite conclusions can be drawn.

Finally, a remark should be made with regard to the protein content of plant-based diets. Even though protein is not considered a nutrient of prime interest for the ageing brain, adequate consumption of high-quality protein is crucial for the ageing muscle and the prevention of sarcopenia [35]. Consuming a plant-based diet increases the risk of inadequate protein intake, due to the lower protein density and suboptimal essential amino acid content of plant foods [36]. Therefore, caution is warranted before advising older adults to reduce their intake of animal-based products.

In conclusion, we did not demonstrate a beneficial association of better adherence to a plant-based diet with cognitive ageing, which could be due to the lower intakes of vitamin B12, DHA and EPA in individuals with higher plant-based diet adherence. Possibly, such association between plant-based diet adherence and cognition exists in a subpopulation of fish-consumers with a fish intake of at least one portion per week. This would be in line with earlier findings that plant-centred diets that include regular fish consumption, such as the MedDiet and MIND diet, may offer benefits for the ageing brain.

## Supplementary Information

Below is the link to the electronic supplementary material.

Supplementary file1 (DOCX 36 KB)

## Data Availability

Data and code is available upon reasonable request in consultation with the study team.

## Abbreviations

BMI: Body Mass Index
B-proof: B-vitamins for the Prevention of Osteoporotic Fractures
DHA: Docosahexaenoic acid
EPA: Eicosapentaenoic acid
FFQ: Food frequency questionnaire
MedDiet: Mediterranean diet
MIND: Mediterranean-Dietary Approaches to Stop Hypertension Intervention for Neurodegenerative Delay
MMSE: Mini Mental State Examination
RAVLT: Rey Auditory Verbal Learning Test
SDMT: Symbol Digit Modalities Test
TICS: Telephone Interview of Cognitive Status
TMT: Trail Making Test
