# Supplementary material for: The association between adherence to a plant-based diet and cognitive ageing
Source: Eur J Nutr. 2023 Mar 11;62(5):2053–62. doi: 10.1007/s00394-023-03130-y (PMC10349692; doi:10.1007/s00394-023-03130-y)
Supplement: Supplementary file 1 — Supplementary file1 (DOCX 36 KB) [file 394_2023_3130_MOESM1_ESM.docx]

**Supplementary information**

**The association between adherence to a plant-based diet and cognitive ageing**

Annick P.M. van Soest^1^ (ORCID: 0000-0003-1702-1345), Ondine van de Rest^1^ (ORCID: 0000-0002-4632-7418), Renger F. Witkamp^1^ (ORCID: 0000-0002-7935-8261), Nathalie van der Velde^2 3^(ORCID: 0000-0002-6477-6209), Lisette C.P.G.M. de Groot^1^(ORCID: 0000-0003-2778-2789)

^1^ Division of Human Nutrition and Health, Wageningen University & Research, Wageningen, The Netherlands

^2^ Department of Internal Medicine, Section of Geriatric Medicine, Amsterdam UMC, University of Amsterdam, Amsterdam, The Netherlands

^3^ Amsterdam Public Health, Aging and Later Life, Amsterdam, The Netherlands.

Corresponding author: Annick van Soest, P.O. Box 12 6700 AA Wageningen The Netherlands, +31 317 488 077, annick.vansoest@wur.nl

**Supplementary table 1**: Overview of food items constituting the 18 food groups.

| Plant food groups | | |
| --- | --- | --- |
| *Healthy* | | |
|  | Whole grains | Whole grain breakfast cereal, cooked oatmeal, wheat porridge, whole grain rusk, whole grain crispbread, rye bread, whole grain bread, whole grain pasta, brown rice, bulgur, millet, couscous |
|  | Fruits | Apple, banana, orange, strawberry, other fruits |
|  | Vegetables | Cauliflower, broccoli and other cabbages, spinach, beets, endive, green beans, other cooked vegetables, lettuce, raw endive, other raw vegetables |
|  | Nuts | Peanut butter, peanuts, cocktail nuts, walnuts, mixed nuts, other nuts and seeds |
|  | Legumes | Legumes, soy products |
|  | Vegetable oils | Olive oil, dressing based on oil, other oils |
|  | Tea & coffee | Tea, coffee |
| *Less healthy* | | |
|  | Fruit juices | Orange juice, other juices |
|  | Refined grains | Cornflakes, white rusk, rice waffles, cream crackers, croissants, white bread, raisin bread, gingerbread, white pasta, white rice |
|  | Potatoes | Fries, chips, cooked and baked potatoes, mashed potatoes |
|  | Sugar sweetened beverages | Regular soft drinks, light soft drinks |
|  | Sweets and desserts | Sweet bread toppings, sugar, cookies, cake, chocolate, candy bars, candy, water ice |
| Animal food groups | | |
|  | Animal fat | Butter, lard |
|  | Dairy | Milk, buttermilk, chocolate milk, yoghurt, custard, drink breakfast, cheese, cream, ice cream, |
|  | Egg | Eggs |
|  | Fish and seafood | Shellfish, mussels, flounder, trout, herring, salmon, other types of fish |
|  | Meat | Liver, ham, bacon, chicken, turkey, minced meat, beef, pork, organ meats, smoked sausage, fried meat snacks |
|  | Misc. animal-based food | Pizza, pancakes, creamy salad dressings, mayonnaise, fried spring roll, meat/fish salads |

**Supplementary table 2**: Description of cognitive tests

| Domain | Test | Description | Scoring |
| --- | --- | --- | --- |
| Episodic memory | RAVLT immediate | Recall of 15 words in five trials | 0-45 |
|  | RAVLT delayed | Delayed recall of the 15 words after 20 minutes | 0-15 |
|  | RAVLT recognition | Recognition of the 15 words in a list of 30 words | 0-30 |
| Attention & Working memory | Digit span forward | Recall of digit sequences with increasing length in forward order | 0-9 |
|  | Digit span backward | Recall of digit sequences with increasing length in backward order | 0-8 |
| Information processing speed | Stroop part I and II | Naming colour words written in black ink (part I) and coloured blocks (part II) as fast as possible.  Outcome is mean part I and II | 0 – ∞ s |
|  | Trail making test part A | Draw lines connecting numbers in chronological order as fast as possible | 0 - 300 s |
|  | SDMT | Match symbols with digits within 90 s as fast as possible. | 0 - 110 |
| Executive functioning | Stroop interference | Naming colour words written in black ink (part I), coloured blocks (part II) and colour words written in an incongruent colour ink (part III) Outcome is part III corrected by parts I and II | 0 – ∞ |
|  | Trail making test part B/A | Draw lines connecting numbers in chronological order (part A) or numbers and letters alternating in chronological and alphabetical order (part B). Outcome is ratio part B/A | 0-300 s |
|  | Letter fluency | Name as many words as possible starting with a specific letter in 60 s | 0 - ∞ |

Abbreviations: Rey Auditory Verbal Learning Test (RAVLT); Symbol Digit Modalities Test (SDMT)

| Nutrient | Total  (n=658) | Tertile 1  (n=226) | Tertile 2 (n=202) | Tertile 3 (n=230) | p-value |
| --- | --- | --- | --- | --- | --- |
| Energy (kCal) | 1945 ± 508 | 1981 ± 505 | 1863 ± 530 | 1980 ± 483 | 0.02 |
| Protein (g) | 73 ± 18 | 77 ± 18 | 71 ± 19 | 71 ± 16 | <0.01 |
| Protein, animal origin (g) | 45 ± 13 | 51 ± 14 | 44 ± 13 | 41 ± 11 | < 0.001 |
| Protein, plant origin (g) | 28 ± 8 | 26 ± 8 | 27 ± 8 | 31 ± 8 | < 0.001 |
| Carbohydrates (g) | 214 ± 62 | 202 ± 61 | 204 ± 62 | 234 ± 59 | < 0.001 |
| Sugar (g) | 111 ± 40 | 105 ± 42 | 105 ± 38 | 122 ± 38 | < 0.001 |
| Starch (g) | 102 ± 32 | 97 ± 31 | 98 ± 32 | 111 ± 29 | < 0.001 |
| Fibre (g) | 23 ± 7 | 21 ± 6 | 23 ± 7 | 26 ± 7 | < 0.001 |
| Fat (g) | 78 ± 27 | 83 ± 28 | 74 ± 29 | 76 ± 25 | <0.01 |
| Cholesterol (mg) | 202 ± 80 | 246 ± 90 | 194 ± 71 | 167 ± 53 | < 0.001 |
| SFA (g) | 28 ± 11 | 32 ± 14 | 27 ± 10 | 26 ± 8 | < 0.001 |
| MUFA (g) | 26 ± 10 | 28 ± 9 | 25 ± 10 | 27 ± 10 | 0.03 |
| PUFA (g) | 16 ± 8 | 16 ± 7 | 15 ± 8 | 16 ± 7 | 0.09 |
| Alcohol (g) | 14 ± 14 | 17 ± 16 | 14 ± 13 | 10 ± 11 | < 0.001 |
| linoleic acid (g) | 13 ± 7 | 13 ± 6 | 12 ± 7 | 14 ± 7 | 0.05 |
| α-linolenic acid (g) | 1.3 ± 0.7 | 1.3 ± 0.7 | 1.2 ± 0.8 | 1.4 ± 0.7 | 0.11 |
| EPA (g) | 0.07 ± 0.08 | 0.08 ± 0.08 | 0.07 ± 0.07 | 0.06 ± 0.08 | 0.01 |
| DHA (g) | 0.11 ± 0.11 | 0.13 ± 0.12 | 0.11 ± 0.10 | 0.10 ± 0.12 | 0.01 |
| Vitamin B12 (mg) | 4.1 ± 2.0 | 4.8 ± 2.3 | 4.0 ± 1.9 | 3.5 ± 1.4 | < 0.001 |
| Folic acid (mcg) | 187 ± 55 | 180 ± 52 | 185 ± 60 | 197 ± 52 | <0.01 |

**Supplementary table 3**: Nutrient intake according to overall plant-based diet index tertile

Abbreviations: SFA: saturated fatty acids, MUFA: monounsaturated fatty acids, PUFA: polyunsaturated fatty acids, EPA: eicosapentaenoic acid, DHA: docosahexaenoic acid. Data are mean ± SD

|  | PDI | | | hPDI | | | uPDI | | |
| --- | --- | --- | --- | --- | --- | --- | --- | --- | --- |
|  | **Crude model** | **Model 1** | **Model 2** | **Crude model** | **Model 1** | **Model 2** | **Crude model** | **Model 1** | **Model 2** |
| Episodic memory | | | | | | | | | |
| Tertile 1 | REF | REF | REF | REF | REF | REF | REF | REF | REF |
| Tertile 2 | 0.09 [-0.05, 0.22] 0.20 | 0.03 [-0.09, 0.16] 0.61 | 0.06 [-0.06, 0.19] 0.33 | 0.00 [-0.13, 0.13] 0.95 | -0.04 [-0.16, 0.08] 0.54 | -0.03 [-0.16, 0.09] 0.58 | -0.07 [-0.20, 0.06] 0.32 | -0.02 [-0.15, 0.10] 0.70 | -0.02 [-0.14, 0.11] 0.80 |
| Tertile 3 | 0.16 [0.03, 0.29] 0.02 | 0.12 [0.00, 0.24] 0.06 | 0.16 [0.03, 0.28] 0.01 | 0.09 [-0.04, 0.22] 0.17 | 0.03 [-0.10, 0.15] 0.67 | 0.01 [-0.12, 0.13] 0.91 | -0.16 [-0.29, -0.03] 0.02 | -0.01 [-0.14, 0.12] 0.90 | 0.03 [-0.10, 0.16] 0.67 |
| Continuous | 0.08 [-0.01, 0.16] 0.08 | 0.05 [-0.03, 0.13] 0.19 | 0.07 [-0.01, 0.16] 0.08 | 0.07 [-0.01, 0.14] 0.10 | 0.01 [-0.06, 0.09] 0.76 | 0.00 [-0.08, 0.07] 0.91 | -0.09 [-0.17, -0.01] 0.03 | 0.00 [-0.08, 0.08] 1.00 | 0.03 [-0.05, 0.11] 0.46 |
| Attention & working memory | | | | | | | | | |
| Tertile 1 | REF | REF | REF | REF | REF | REF | REF | REF | REF |
| Tertile 2 | 0.14 [-0.02, 0.30] 0.09 | 0.08 [-0.07, 0.23] 0.30 | 0.09 [-0.06, 0.25] 0.24 | 0.08 [-0.08, 0.24] 0.31 | 0.06 [-0.09, 0.21] 0.45 | 0.05 [-0.10, 0.20] 0.48 | -0.01 [-0.18, 0.15] 0.86 | 0.05 [-0.11, 0.20] 0.56 | 0.03 [-0.12, 0.19] 0.69 |
| Tertile 3 | -0.11 [-0.27, 0.05] 0.18 | -0.12 [-0.27, 0.03] 0.11 | -0.11 [-0.26, 0.05] 0.17 | 0.00 [-0.16, 0.16] 0.99 | -0.09 [-0.25, 0.06] 0.22 | -0.10 [-0.25, 0.06] 0.22 | -0.09 [-0.26, 0.07] 0.27 | 0.03 [-0.12, 0.19] 0.68 | 0.03 [-0.14, 0.19] 0.74 |
| Continuous | -0.02 [-0.12, 0.09] 0.75 | -0.03 [-0.13, 0.07] 0.51 | -0.02 [-0.12, 0.08] 0.69 | 0.01 [-0.09, 0.11] 0.84 | -0.06 [-0.15, 0.03] 0.20 | -0.06 [-0.15, 0.03] 0.22 | -0.06 [-0.16, 0.04] 0.22 | 0.01 [-0.09, 0.10] 0.87 | 0.01 [-0.09, 0.10] 0.91 |
| Information processing speed | | | | | | | | | |
| Tertile 1 | REF | REF | REF | REF | REF | REF | REF | REF | REF |
| Tertile 2 | 0.08 [-0.07, 0.23] 0.32 | 0.06 [-0.08, 0.20] 0.40 | 0.08 [-0.06, 0.22] 0.28 | 0.07 [-0.08, 0.22] 0.37 | 0.05 [-0.09, 0.18] 0.49 | 0.06 [-0.08, 0.19] 0.41 | -0.07 [-0.23, 0.08] 0.34 | -0.06 [-0.20, 0.08] 0.39 | -0.05 [-0.19, 0.09] 0.48 |
| Tertile 3 | 0.05 [-0.10, 0.20] 0.50 | 0.06 [-0.08, 0.19] 0.42 | 0.05 [-0.09, 0.19] 0.46 | 0.08 [-0.07, 0.23] 0.31 | -0.01 [-0.15, 0.13] 0.91 | -0.04 [-0.18, 0.10] 0.56 | -0.15 [-0.30, 0.00] 0.06 | -0.05 [-0.19, 0.09] 0.50 | -0.02 [-0.16, 0.13] 0.80 |
| Continuous | 0.06 [-0.04, 0.16] 0.22 | 0.05 [-0.04, 0.14] 0.32 | 0.04 [-0.05, 0.13] 0.39 | 0.05 [-0.04, 0.14] 0.26 | 0.00 [-0.08, 0.08] 0.97 | -0.03 [-0.11, 0.06] 0.51 | -0.07 [-0.16, 0.02] 0.12 | -0.02 [-0.10, 0.07] 0.67 | 0.00 [-0.09, 0.09] 0.97 |
| Executive functioning | | | | | | | | | |
| Tertile 1 | REF | REF | REF | REF | REF | REF | REF | REF | REF |
| Tertile 2 | 0.15 [0.01, 0.28] 0.04 | 0.09 [-0.03, 0.22] 0.15 | 0.11 [-0.02, 0.24] 0.09 | 0.08 [-0.06, 0.21] 0.26 | 0.04 [-0.08, 0.17] 0.51 | 0.04 [-0.09, 0.16] 0.53 | 0.02 [-0.12, 0.15] 0.79 | 0.05 [-0.08, 0.18] 0.44 | 0.04 [-0.08, 0.17] 0.50 |
| Tertile 3 | 0.07 [-0.06, 0.21] 0.27 | 0.06 [-0.07, 0.18] 0.37 | 0.06 [-0.07, 0.19] 0.34 | 0.12 [-0.01, 0.25] 0.08 | 0.02 [-0.10, 0.15] 0.72 | -0.01 [-0.13, 0.12] 0.93 | -0.13 [-0.27, 0.00] 0.06 | -0.02 [-0.15, 0.11] 0.75 | -0.02 [-0.16, 0.11] 0.76 |
| Continuous | 0.08 [-0.01, 0.17] 0.09 | 0.06 [-0.02, 0.14] 0.15 | 0.06 [-0.02, 0.15] 0.15 | 0.09 [0.01, 0.17] 0.03 | 0.03 [-0.05, 0.10] 0.49 | 0.01 [-0.07, 0.08] 0.90 | -0.10 [-0.18, -0.02] 0.02 | -0.03 [-0.11, 0.04] 0.38 | -0.03 [-0.12, 0.05] 0.40 |

**Supplementary table 4**: Regression output energy-adjusted overall, healthful and unhealthful plant based diet index and domain specific cognitive functioning (cross-sectional)

Abbreviations: PDI; plant-based diet index, hPDI; healthful plant-based diet index, uPDI; unhealthful plant-based diet index.
Model 1: adjusted for age, gender and education. Model 2: additionally adjusted for BMI, physical activity, smoking, alcohol consumption and margarine consumption. Longitudinal analysis was additionally adjusted for baseline cognition score. Data are β [95% CI] p-value. In the continuous analysis, β is shown per 10 points increment in plant-based diet index.

|  | PDI | | | hPDI | | | uPDI | | |
| --- | --- | --- | --- | --- | --- | --- | --- | --- | --- |
|  | **Crude model** | **Model 1** | **Model 2** | **Crude model** | **Model 1** | **Model 2** | **Crude model** | **Model 1** | **Model 2** |
| Episodic memory | | | | | | | | | |
| Tertile 1 | REF | REF | REF | REF | REF | REF | REF | REF | REF |
| Tertile 2 | -0.05 [-0.21, 0.11] 0.53 | -0.06 [-0.21, 0.10] 0.48 | -0.07 [-0.23, 0.10] 0.42 | 0.02 [-0.13, 0.17] 0.80 | 0.01 [-0.14, 0.17] 0.86 | 0.01 [-0.15, 0.16] 0.92 | -0.08 [-0.23, 0.08] 0.32 | -0.07 [-0.22, 0.09] 0.39 | -0.05 [-0.21, 0.11] 0.54 |
| Tertile 3 | -0.04 [-0.19, 0.11] 0.61 | -0.03 [-0.18, 0.13] 0.72 | -0.06 [-0.22, 0.10] 0.49 | -0.02 [-0.17, 0.14] 0.84 | -0.02 [-0.18, 0.13] 0.76 | -0.04 [-0.20, 0.12] 0.64 | -0.07 [-0.22, 0.09] 0.41 | -0.03 [-0.19, 0.13] 0.71 | -0.03 [-0.19, 0.14] 0.76 |
| Continuous | -0.04 [-0.14, 0.06] 0.44 | -0.04 [-0.14, 0.07] 0.47 | -0.06 [-0.17, 0.05] 0.27 | 0.02 [-0.08, 0.11] 0.75 | 0.01 [-0.09, 0.11] 0.83 | 0.00 [-0.10, 0.10] 0.97 | -0.06 [-0.16, 0.03] 0.21 | -0.04 [-0.14, 0.05] 0.39 | -0.04 [-0.14, 0.06] 0.47 |
| Attention & working memory | | | | | | | | | |
| Tertile 1 | REF | REF | REF | REF | REF | REF | REF | REF | REF |
| Tertile 2 | -0.17 [-0.34,-0.01] 0.04 | -0.18 [-0.36,-0.01] 0.03 | -0.20 [-0.37,-0.02] 0.03 | 0.00 [-0.16, 0.17] 0.97 | -0.01 [-0.18, 0.15] 0.89 | 0.00 [-0.17, 0.16] 0.96 | 0.00 [-0.17, 0.17] 0.99 | 0.00 [-0.17, 0.17] 0.98 | 0.01 [-0.16, 0.18] 0.92 |
| Tertile 3 | 0.08 [-0.09, 0.24] 0.35 | 0.06 [-0.10, 0.23] 0.47 | 0.05 [-0.13, 0.22] 0.59 | 0.25 [0.08, 0.42] <0.01 | 0.22 [0.05, 0.39] 0.01 | 0.23 [0.05, 0.41] 0.01 | -0.21 [-0.38, -0.04] 0.02 | -0.19 [-0.36,-0.01] 0.04 | -0.18 [-0.36, -0.01] 0.04 |
| Continuous | 0.06 [-0.05, 0.17] 0.29 | 0.05 [-0.06, 0.16] 0.36 | 0.04 [-0.08, 0.16] 0.48 | 0.15 [0.05, 0.26] <0.01 | 0.14 [0.04, 0.24] 0.01 | 0.14 [0.03, 0.25] 0.01 | -0.17 [-0.27, -0.06] <0.01 | -0.16 [-0.27,-0.06] <0.01 | -0.16 [-0.27, -0.05] <0.01 |
| Information processing speed | | | | | | | | | |
| Tertile 1 | REF | REF | REF | REF | REF | REF | REF | REF | REF |
| Tertile 2 | -0.15 [-0.28,-0.01] 0.04 | -0.13 [-0.26, 0.00] 0.05 | -0.13 [-0.26, 0.01] 0.06 | 0.03 [-0.10, 0.16] 0.65 | 0.04 [-0.09, 0.17] 0.55 | 0.04 [-0.08, 0.17] 0.50 | -0.07 [-0.21, 0.06] 0.29 | -0.07 [-0.20, 0.06] 0.29 | -0.08 [-0.22, 0.05] 0.21 |
| Tertile 3 | -0.08 [-0.21, 0.05] 0.21 | -0.08 [-0.21, 0.05] 0.21 | -0.11 [-0.25, 0.02] 0.09 | 0.06 [-0.07, 0.20] 0.35 | 0.04 [-0.10, 0.17] 0.60 | 0.00 [-0.13, 0.14] 0.99 | -0.06 [-0.20, 0.07] 0.36 | -0.04 [-0.17, 0.10] 0.58 | -0.07 [-0.21, 0.06] 0.30 |
| Continuous | -0.04 [-0.13, 0.05] 0.36 | -0.05 [-0.14, 0.03] 0.23 | -0.07 [-0.16, 0.01] 0.10 | 0.05 [-0.03, 0.13] 0.25 | 0.03 [-0.05, 0.12] 0.40 | 0.01 [-0.07, 0.09] 0.80 | -0.05 [-0.13, 0.03] 0.21 | -0.05 [-0.13, 0.03] 0.24 | -0.07 [-0.16, 0.01] 0.09 |
| Executive functioning | | | | | | | | | |
| Tertile 1 | REF | REF | REF | REF | REF | REF | REF | REF | REF |
| Tertile 2 | 0.09 [-0.04, 0.22] 0.17 | 0.09 [-0.03, 0.22] 0.15 | 0.09 [-0.04, 0.22] 0.15 | -0.01 [-0.13, 0.12] 0.90 | -0.02 [-0.14, 0.11] 0.80 | -0.01 [-0.14, 0.11] 0.81 | 0.02 [-0.11, 0.14] 0.78 | 0.02 [-0.10, 0.15] 0.74 | 0.02 [-0.10, 0.15] 0.72 |
| Tertile 3 | 0.03 [-0.09, 0.16] 0.60 | 0.04 [-0.08, 0.16] 0.56 | 0.01 [-0.11, 0.14] 0.82 | 0.07 [-0.06, 0.20] 0.28 | 0.04 [-0.08, 0.17] 0.50 | 0.01 [-0.12, 0.14] 0.84 | 0.05 [-0.08, 0.18] 0.44 | 0.08 [-0.05, 0.21] 0.21 | 0.06 [-0.07, 0.20] 0.34 |
| Continuous | 0.05 [-0.03, 0.13] 0.26 | 0.04 [-0.04, 0.12] 0.30 | 0.03 [-0.06, 0.11] 0.52 | 0.03 [-0.05, 0.11] 0.47 | 0.01 [-0.06, 0.09] 0.70 | -0.01 [-0.09, 0.07] 0.84 | 0.03 [-0.05, 0.11] 0.47 | 0.04 [-0.04, 0.12] 0.28 | 0.03 [-0.05, 0.12] 0.40 |

**Supplementary table 5**: Regression output energy-adjusted overall, healthful and unhealthful plant based diet index and domain specific cognitive change (longitudinal)

Abbreviations: PDI; plant-based diet index, hPDI; healthful plant-based diet index, uPDI; unhealthful plant-based diet index.
Model 1: adjusted for age, gender and education. Model 2: additionally adjusted for BMI, physical activity, smoking, alcohol consumption and margarine consumption. Longitudinal analysis was additionally adjusted for baseline cognition score. Data are β [95% CI] p-value. In the continuous analysis, β is shown per 10 points increment in plant-based diet index.
